# Data supporting attempted caveolae-mediated phagocytosis of surface-fixed micro-pillars by human osteoblasts

**DOI:** 10.1016/j.dib.2016.02.023

**Published:** 2016-02-17

**Authors:** Caroline Moerke, Petra Mueller, Barbara Nebe

**Affiliations:** a University Medical Center Rostock, Department of Cell Biology, Rostock, Germany

## Abstract

The provided data contains the phagocytic interaction of human MG-63 osteoblasts with micro-particles 6 µm in size as well as geometric micro-pillared topography with micro-pillar sizes 5 µm of length, width, height and spacing respectively related to the research article entitled “Attempted caveolae-mediated phagocytosis of surface-fixed micro-pillars by human osteoblasts” in the *Biomaterials* journal. [1] Micro-particle treatment was used as positive control triggering phagocytosis by the osteoblasts. Caveolin-1 (Cav-1) as major structural component of caveolae [2] plays an important role in the phagocytic process of micro-particles and -pillars. Data related to the experiments in [1] with siRNA-mediated knockdown are presented here as well as micro-particle control experiments, tubulin analysis on the micro-pillared topography and initial cell interaction with the micro-pillars.

Specifications table

**Table:** 

Subject area	*Biology*
More specific subject area	*Osteoblast interaction with biomaterial topographies*
Type of data	*Images, Movie*
How data was acquired	*Confocal Laser Scanning Microscope (LSM780; Carl Zeiss), SEM (DSM910A; Carl Zeiss)*
Data format	*Raw data*
Experimental factors	*Effect of micro-pillared topography and micro-particles on osteoblast cell behavior*
Experimental features	*Visualization of protein localization changes via immuno-labeling after particle treatment and on the micro-pillared topography; cell morphology changes after micro-particles treatment*
Data source location	*University Medical Center Rostock, Germany*
Data accessibility	*Data is available in this article and related to*[1]

Value of the data

• The data inform future studies of topography-induced phagocytic responses of osteoblasts, which is of relevance for designing new implant surfaces.
• Micro-pillared topography has an enormous effect on the actin arrangement but no impact on tubulin cytoskeleton organization, thus the data inform about the complexity of cellular reactions on biomaterial topographies.
• Utilizing 6 µm sized particles showed triggering phagocytosis in osteoblasts with CD68 involvement and only partial Caveolin-1 dependency relevant for researches in the implant wear-debris area.
• The data displays the independence of Caveolin-1 on actin reorganization during phagocytosis.

## 1 Data, experimental design, materials and methods

### 1.1 Micro-particle treatment of human MG-63 cells and SEM sample preparation

MG-63 (American Type Culture Collection ATCC®, CRL-1427) were grown in Dulbecco’s modified eagle medium (DMEM, Life Technologies GmbH, Darmstadt, Germany) with 10% fetal calf serum (FCS) (Biochrom FCS Superior, Merck KGaA, Darmstadt, Germany) and 1% gentamycin (Ratiopharm GmbH, Ulm, Germany) at 37 °C in a humidified atmosphere with 5% CO_2_. Cells were seeded on cover glasses and incubated for 1 h at 37 °C and 5% CO_2_ to ensure adhesion. Afterwards the cells were incubated with melamine particles 6 µm in size marked with FITC (Sigma Aldrich) in a concentration of 10^5^ ml^−1^ for 24 h. For SEM sample preparations cells were washed with PBS three times and then fixed with 2.5% glutardialdehyde (Merck KGaA) for 1 h at RT, dehydrated through a graded series of ethanol (30%, 50%, 75%, 90% and 100% for 5, 5, 15, 10 min and twice for 10 min, respectively) dried in a critical point dryer (K 850, EMITECH, Taunusstein, Germany) and then samples were sputtered with gold for 100 s (layer ca. 20 nm) (SCD 004, BAL-TEC, Wetzlar, Germany).

### 1.2 Immunofluorescence staining

Osteoblastic cells were cultured on the Ti arrays described in [1], [3] and after micro-particle treatment for 24 h, washed three times with PBS and then fixed with 4% paraformaldehyde (PFA) (10 min; room temperature, RT) (Sigma-Aldrich). After washing thrice with PBS, the cells were permeabilized with 0.1% Triton X-100 (10 min, RT) (Merck), washed again three times with PBS and blocked with 2% bovine serum albumin (BSA) (Sigma-Aldrich) in PBS (30 min, RT). For actin filament staining, cells were incubated with phalloidine coupled with tetramethyl-rhodamine (TRITC) (5 µg/ml in PBS) (Sigma-Aldrich). The following primary antibodies (diluted in PBS) were used for the immunolabeling at RT for 1 h: polyclonal rabbit anti-caveolin-1 (New England Biolabs GmbH) (1:400), polyclonal rabbit anti-CD68 (Proteintech Europe Inc.) (1:25), monoclonal mouse anti-α tubulin (1:50). Secondary antibodies anti-rabbit-IgG-AF488, anti-mouse-IgG-AF488 and anti-rabbit-IgG-AF546 (Life Technologies, diluted 1:200 in PBS) were applied for 30 min at RT. The samples were embedded with fluoroshield mounting media (Sigma-Aldrich). Image acquisition was performed with the ZEISS oil immersion objective (C-Apochromat63) and the ZEN 2011 (black version) software (Carl Zeiss AG). Images were displayed as three dimensional (3D) z-stacks (13 stacks with an interval of 1 µm) in addition with a 2D *xz*- and *yz*-plane at micro-particle experiments.

### 1.3 Cav-1 transfection

Small interfering RNA (siRNA) against Cav-1 as well as control siRNA were obtained from Ambion (Life Technologies GmbH). For the transfection, 30,000 MG-63 cells were seeded in a 24-well plate and cultured overnight. Then the cells were transfected with 50 nM siRNA using MG-63 Transfection Reagent (Altogen Biosystems, Las Vegas, NV, USA) according to the manufacturer’s instructions. 48 h after the transfection the cells were ready for further experiments. For once they were treated for 24 h with 6 µm particles and they were also trypsinated and seeded onto the Ti arrays for 24 h.

### 1.4 Live cell imaging

For the observation of actin in living cells, the GFP-actin baculovirus expression vector (CellLight™ Actin-GFP BacMam 2.0, Life Technologies) was transfected into MG-63 cells according to the manufacturer’s protocol. Cells were cultured for over 24 h to examine GFP-actin expression. Afterwards the cells were trypsinized and seeded onto the Ti arrays for 15 min to ensure adhesion. Then the Ti arrays were placed into an IBIDI µ-Dish 35 mm high (Ibidi LCC) with the adherent cells towards the bottom of the dish containing 2 ml of DMEM. The actin dynamics of the vital cells was visualized with the inverted confocal laser scanning microscope using a 20× (EC Plan-Neofluar) objective (Carl Zeiss AG) under incubation at 37 °C and 5% CO_2_. Thus actin dynamics were visualized with cultivation against gravity so the cells hanging upside down only secured by the adhesion. Image acquisition was every 10 min for 7 h and converted into a video via the ZEN 2011 (black version) software.

## 2 Data

### 2.1 Micro-particle uptake and distribution

**Fig. 1:**
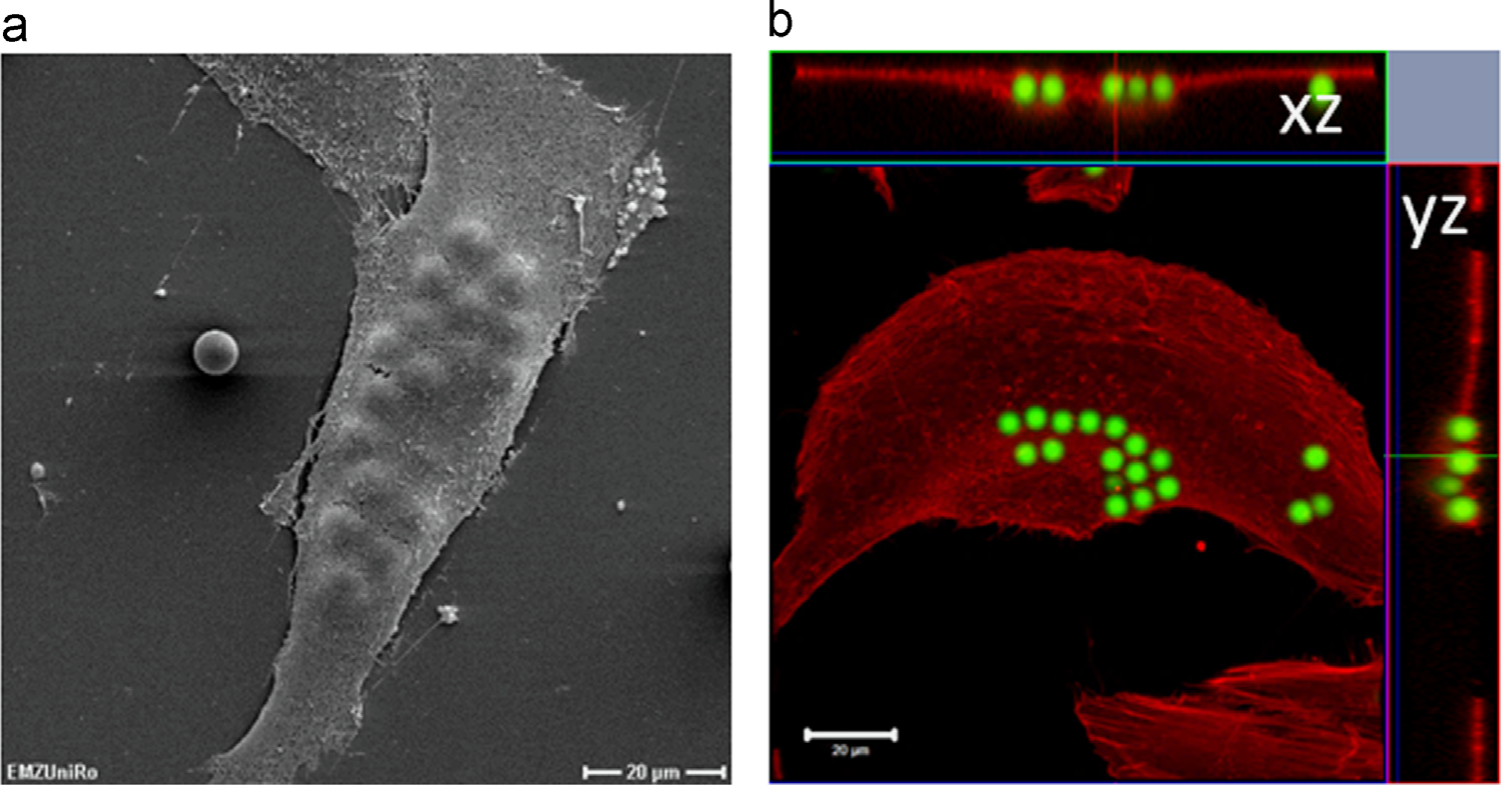
6 µm particle distributions after phagocytosis in human MG-63 cells. (A) cell morphology visualized by SEM (1000× magnification, bar 20 µm) and (B) actin fluorescent labeling displaying 3D z-stack image with confocal *xz*-plane (above) and *yz*-plane (right); bar 20 µm.

In Fig. 1 the cell morphology and actin cytoskeleton organization of human MG-63 cells after micro-particle treatment is presented. The cells phagocytize several micro-particles during 24 h incubation time. All particles were concentrated and not freely distributed inside the cells.

### 2.2 Actin cytoskeleton organization after siRNA-mediated Cav-1 knockdown in MG-63 cells after micro-particle treatment and on the micro-pillared topography

**Fig. 2:**
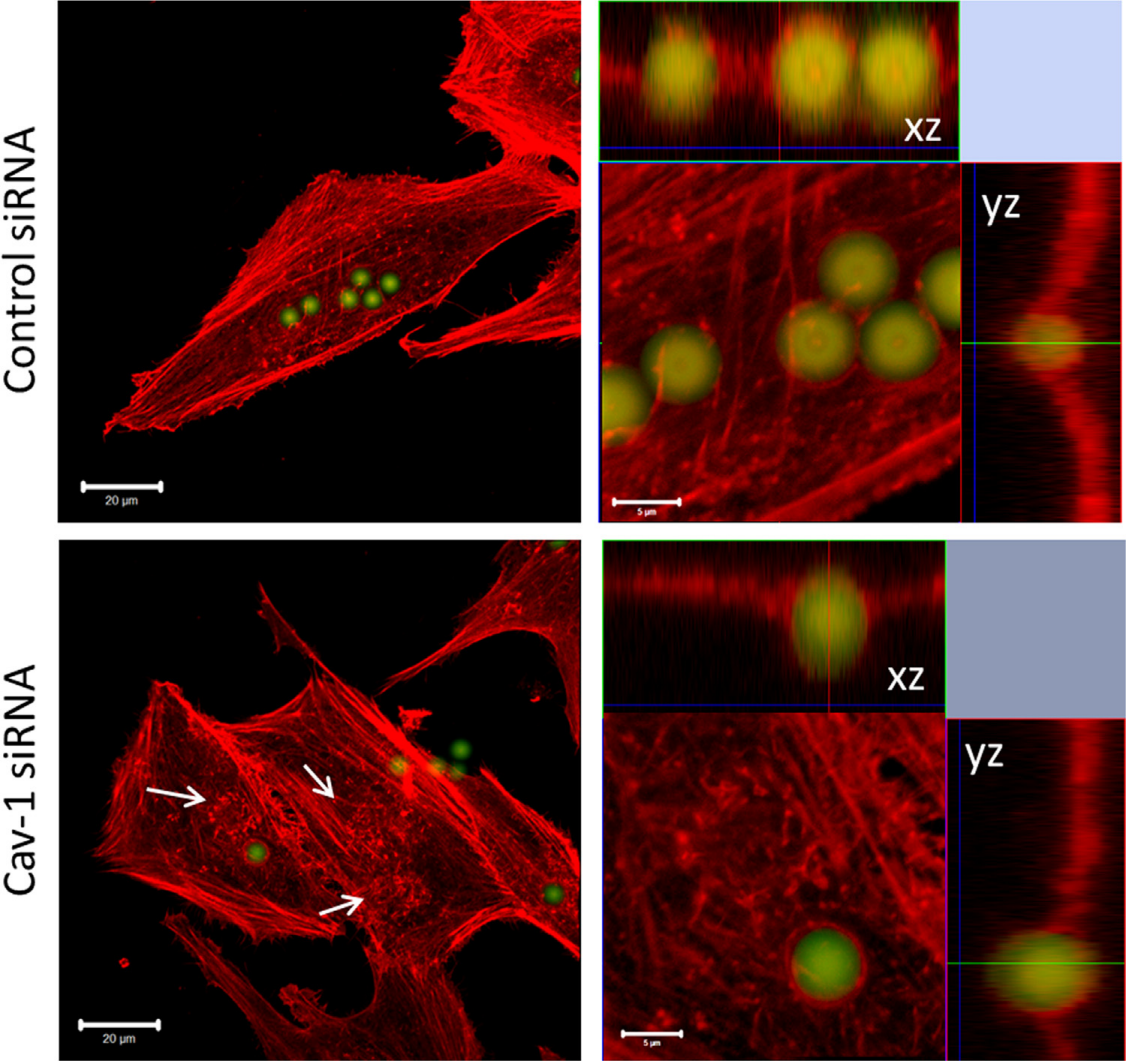
Actin cytoskeleton organization during micro-particle treatment after siRNA-mediated Caveolin-1 (Cav-1) knockdown in MG-63 osteoblasts. Bars 20 µm (left) and 5 µm (right).

The actin cytoskeleton after Cav-1 attenuation was arranged in short filaments around non-internalized particles, which were washed away during the preparation (Fig. 2). The images show a reduced particle phagocytosis by MG-63 cells, but no complete inhibition of the phagocytosis, as reported in the past. [4]

**Fig. 3:**
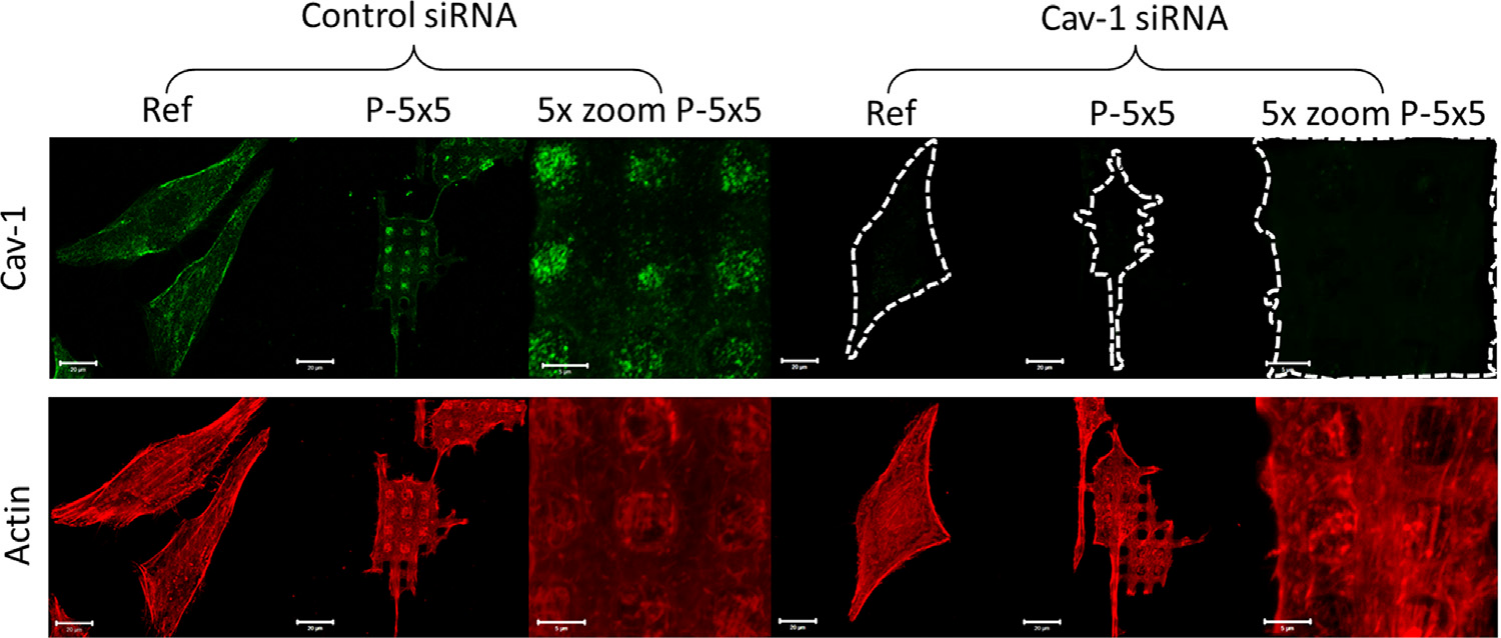
Actin cytoskeleton (red) organization after siRNA mediated Caveolin-1 (Cav-1) knockdown in MG-63 osteoblasts after 24 h on planar reference (Ref) and micro-pillars (P-5×5). For verification of Cav-1 knockdown Cav-1 was immuno-labeled in green, equal setting for the image acquisition, bars 20 µm and for 5× zoom 5 µm.

The MG-63 osteoblasts with siRNA mediated Cav-1 knockdown grown on the micro-pillars indicated the same rearrangement of the actin cytoskeleton as seen in control cells, illustrated by Fig. 3.

### 2.3 CD68 localization after micro-particle phagocytosis

**Fig. 4:**
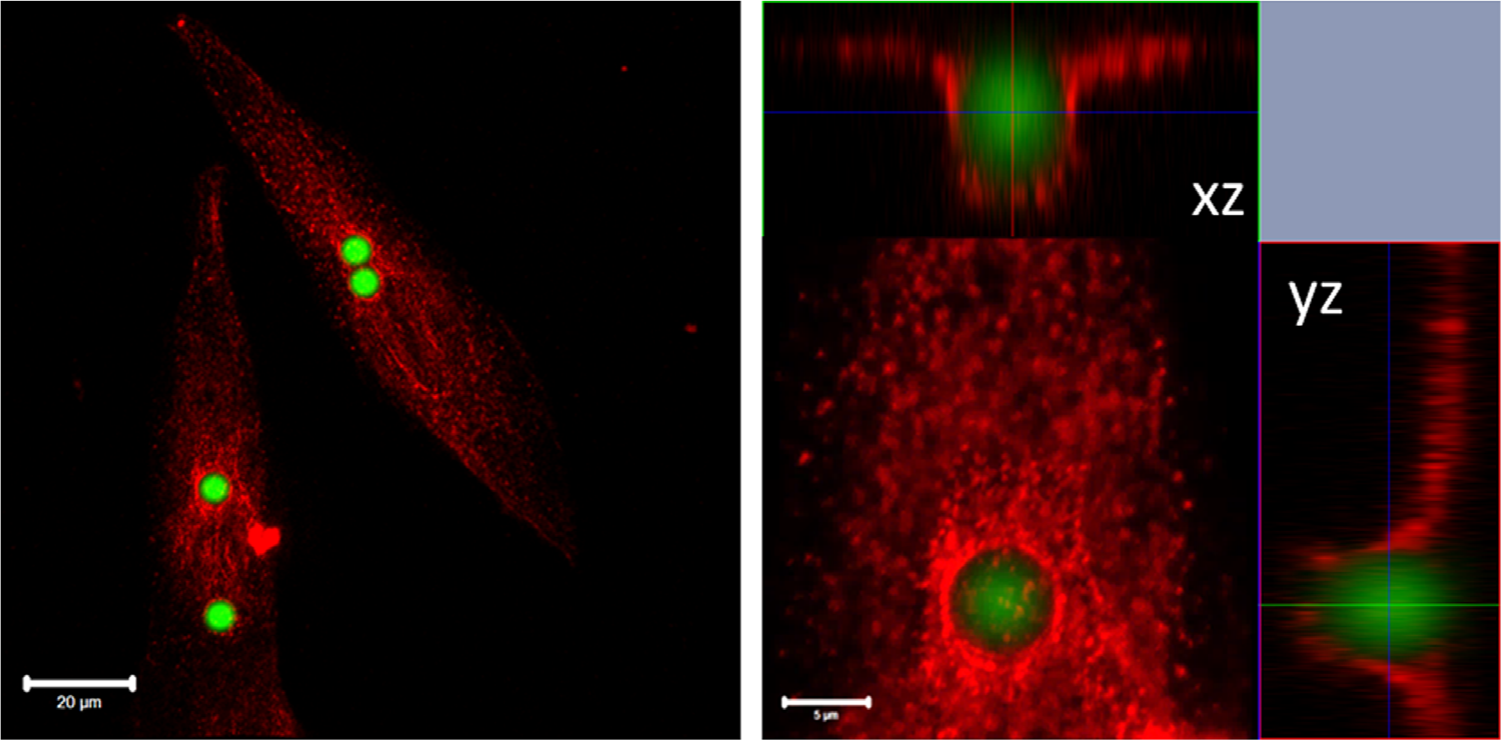
CD68 localization in MG-63 cells treated with 6 µm particles for 24 h. Bar left 20 µm and right 5 µm for 5× zoom.

Immunofluorescence staining showed an enrichment of CD68 around internalized particles 6 µm in size, presented by Fig. 4.

### 2.4 α-Tubulin localization in MG-63 osteoblasts on micro-pillared topography

**Fig. 5:**
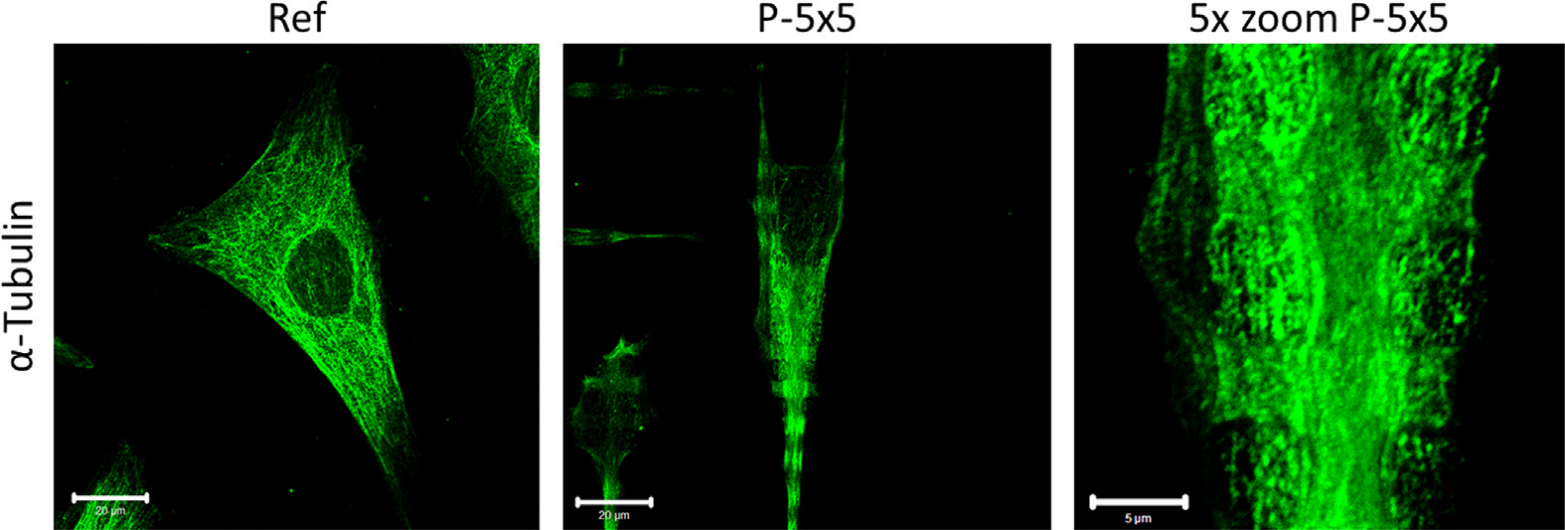
α-Tubulin immuno-labeling of MG-63 osteoblasts after 24 h on the micro-pillars (P-5×5) and the planar reference (Ref) (left and middle bar 20 µm, right 5 µm).

Fig. 5 displayed an unaltered α-Tubulin organization in MG-63 cells grown on the micro-pillared topography.

### 2.5 Initial cell dynamic on the micro-pillared topography

The MG-63 cells are actively testing the underlying topography with their filopodia during the first 6 h after cell seeding onto the micro-pillared topography, shown by Movie 1.

Supplementary material related to this article can be found online at 10.1016/j.dib.2016.02.023.

The following is the Supplementary material related to this article Movie 1, Movie 1.

Movie 1 Live cell imaging of MG−63 osteoblasts on in micro-pillars (P−5×5) during the first 6 h cultivation.
